# Bleeding in neonates with severe thrombocytopenia: a retrospective cohort study

**DOI:** 10.1186/s12887-022-03802-4

**Published:** 2022-12-22

**Authors:** Ting Peng, Yuanyuan Shan, Peng Zhang, Guoqiang Cheng

**Affiliations:** Department of Neonatology, National Children’s Medical Center, Children’s Hospital of Fudan University, Shanghai, China

**Keywords:** Bleeding, Thrombocytopenia, Platelet transfusion, Neonates, Severe

## Abstract

**Background:**

Severe neonatal thrombocytopenia is a rare disease with multiple etiologies. Severe thrombocytopenia with bleeding is life-threatening and has attracted significant attention from clinicians. However, only a few studies have focused on the association between severe thrombocytopenia and bleeding. Thus, this study aimed to describe the neonates’ postnatal age at which severe thrombocytopenia was first recognized, clinical characteristics, bleeding patterns, and outcomes and to evaluate the association between minimum platelet count and bleeding.

**Methods:**

A single-center retrospective cohort study for neonates with severe thrombocytopenia (platelet count ≤ 50 × 10^9^/L) was conducted. Neonates who were admitted to our neonatal intensive care unit between October 2016 and February 2021 and developed severe thrombocytopenia were analyzed. Data were collected retrospectively until the patients were referred to other hospitals, discharged, or deceased.

**Results:**

Among the 5819 neonatal inpatients, 170 with severe thrombocytopenia were included in this study. More than 30% of the patients had severe thrombocytopenia in the first 3 days of life. Among the 118 neonates with bleeding, 47 had more than one type of pathological bleeding. Neonates with very severe thrombocytopenia (point estimate: 53.7%, 95% confidence interval [CI]: 44.2%–63.1%) had a higher incidence rate of cutaneous bleeding than those with severe thrombocytopenia (point estimate: 23.4%, 95% CI: 12.3%–34.4%). The gestational age (median: 36.2 [interquartile range [IQR]: 31.4–39.0] weeks) and birth weight (median: 2310 [IQR: 1213–3210] g) of the major bleeding group were the lowest among no bleeding, minor bleeding, and major bleeding groups. Regression analysis controlled for confounders and confirmed that a lower platelet count (odds ratio [OR]: 2.504 [95% CI: 1.180–5.314], *P* = 0.017) was associated with a significant increase in the rate of bleeding. Very severe thrombocytopenia (point estimate: 49.1%, 95% CI: 39.6%–58.6%) had a higher rate of platelet transfusion than severe thrombocytopenia (point estimate: 5.7%, 95% CI: 0.7%–10.7%). The mortality rate was higher in neonates with bleeding than in those without bleeding (point estimates with 95% CI: 33.1% [24.4%–41.7%] vs. 7.7% [0.2%–15.2%]).

**Conclusions:**

These findings describe the incidence of severe thrombocytopenia and demonstrate that a lower platelet count is associated with an increased bleeding rate in patients with severe thrombocytopenia.

**Supplementary Information:**

The online version contains supplementary material available at 10.1186/s12887-022-03802-4.

## Background

Neonatal thrombocytopenia is defined as a platelet count < 150 × 10^9^/L, with an incidence rate ranging from 1 to 5% in all neonates and up to 35% in all patients admitted to the neonatal intensive care unit (NICU) [1–3]. The incidence of severe thrombocytopenia (platelet count ≤ 50 × 10^9^/L) varies from 2.1% to 10% in related studies [3–7]. Chronic fetal hypoxia is the most common cause of early-onset (first 72 h of life) thrombocytopenia [8, 9]. In contrast, late-onset (after the first 3 days of life) thrombocytopenia is caused by sepsis and/or necrotizing enterocolitis (NEC) [8].

Neonatal thrombocytopenia is relatively mild and not life-threatening. However, neonates with severe thrombocytopenia may have the most serious clinical symptoms and outcomes. In severe thrombocytopenia, the major concern is bleeding, such as intraventricular hemorrhage (IVH), pulmonary hemorrhage, and cutaneous bleeding. Previous studies have investigated the burden and patterns of major and minor bleeding in severe neonatal thrombocytopenia [4, 5, 10]. In a retrospective study, researchers hypothesized a causal association between minimum platelet count and cutaneous bleeding [5]. A prospective study observed major bleeding in neonates with severe thrombocytopenia and explored platelet transfusion (PT) practices [4]. Subsequently, they described the patterns of minor bleeding and established the significance of clinical risk factors for bleeding [10]. More recently, a prediction model was developed to calculate bleeding risk during the first week after the onset of severe thrombocytopenia [11]. Unfortunately, the association between severe thrombocytopenia and bleeding remains controversial and has only been investigated in a limited number of studies. Therefore, the study aimed to determine the postnatal age when severe thrombocytopenia was first diagnosed; to identify the clinical features, bleeding conditions, and outcomes of patients with severe thrombocytopenia; and to explore the association between the lowest platelet count and bleeding.

## Methods

We retrospectively collected the electronic medical records of all neonates admitted to the NICU of the Children’s Hospital of Fudan University between October 2016 and February 2021. The data were collected and processed anonymously.

### Patients

Severe thrombocytopenia, confirmed by a peripheral blood smear, was defined as a platelet count ≤ 50 × 10^9^/L. The cohort comprised all neonates with at least one platelet count ≤ 50 × 10^9^/L. We excluded patients: (i) whose platelet count was suspicious, (ii) undergoing exchange transfusion, (iii) whose clinical data were missing, and (iv) undergoing an operation. First, the degree of severe thrombocytopenia was categorized into severe (platelet count of 31–50 × 10^9^/L) and very severe (platelet count ≤ 30 × 10^9^/L) based on the lowest platelet count during hospitalization to compare bleeding events and determine the association between the lowest platelet count and bleeding [6]. Second, we divided the patients into three subgroups according to the severity of bleeding: no, minor, and major bleeding, to describe their characteristics, comorbidities, and outcomes. We used the following guidelines for PT in our NICU: (i) platelet count ≤ 30 × 10^9^/L and stable; (ii) platelet count ≤ 50 × 10^9^/L and unstable and/or overt bleeding; and (iii) platelet count > 30 × 10^9^/L without serious bleeding, requiring no PT.

### Data collection

Maternal data (mode of delivery, maternal diseases, including diabetes, premature rupture of membrane, preeclampsia, and thrombocytopenia in pregnancy), perinatal data (multiple births, gestational age [GA] in weeks, birth weight [BW] in grams, Apgar score at 1 min and 5 min), asphyxia, and neonatal data (sex, various types of bleeding, patent ductus arteriosus [PDA], bronchopulmonary dysplasia, neonatal respiratory distress syndrome [NRDS], neonatal shock, coagulation disorders, early-onset sepsis [EOS] and late-onset sepsis [LOS], chromosomal disorders, NEC, platelet counts in daily life, onset of severe thrombocytopenia, hospital stay, treatment [PT and mechanical ventilation], outcomes) were collected.

An Apgar score < 7 at 1 and 5 min was considered a low Apgar score. Neonatal asphyxia was diagnosed with an Apgar score < 7 at 1 and 5 min without establishing spontaneous respiration and an umbilical artery pH ≤ 7.15, excluding other causes of low Apgar score in our own policy. Neonatal sepsis was defined as (i) positive blood culture and (ii) nonspecific clinical signs of infection and abnormal hematological test results [12]. Sepsis was divided into EOS (within 72 h of life) and LOS (after 72 h of life). NEC was defined according to Bell criteria [13]. Severe thrombocytopenia before 72 h was defined as early-onset severe thrombocytopenia and after 72 h as late-onset severe thrombocytopenia. Intracranial hemorrhage (ICH) included subdural, subarachnoid, solitary (non-cerebellar) parenchymal, and cerebellar parenchymal hemorrhage and IVH. ICH was detected using cranial ultrasonography, computed tomography, and magnetic resonance imaging. Intraventricular/paraventricular hemorrhage was graded using the method described by Papile et al. [14]. Extracranial hemorrhage (ECH) included cutaneous bleeding (petechiae, ecchymosis, and oozing from venipuncture sites) and umbilical, pulmonary, and gastrointestinal hemorrhage. Major bleeding was defined as pulmonary hemorrhage, gastrointestinal hemorrhage, and ICH, except for IVH (I/II)). Minor bleeding was defined as bleeding other than the major bleeding. Data from all patients were collected until they were transferred to other hospitals, discharged, or deceased. Normalization of thrombocytopenia was defined as a platelet count > 150 × 10^9^/L for at least 2 days.

### Statistical analyses

Statistical analyses were performed using the Statistical Package for the Social Sciences Statistics version 23.0 (IBM Corp. Armonk, NY, USA). Quantitative data are expressed as mean and standard deviation or median with interquartile range (IQR). Qualitative data are presented as numbers and percentages. Binary logistic regression analysis was performed to explore the association between the minimum platelet count and bleeding. Confounding variables were controlled, and adjusted odds ratios (ORs) were calculated. The following variables that were significant (*P* < 0.05) in the univariate analysis and were clinically potential risk factors for bleeding were included in the logistic regression analysis: GA, BW, PDA, mechanical ventilation, and coagulation disorders. Confidence intervals (CIs) were calculated at the 95% level. Post-hoc analysis was performed to evaluate the power using Power and Sample Size Calculations (version 3.1.2). A *P* value < 0.05 was considered to be significant.

## Results

### Total patient population

In total, 5819 neonates were admitted to our NICU during the study period. Severe thrombocytopenia was detected in 194 neonates, of whom 24 were excluded from the cohort because of exchange transfusion (*n* = 3) or surgery (*n* = 21). The incidence of severe thrombocytopenia was 3.33%. Of the 170 enrolled neonates, 82 (48.2%), 35 (20.6%), and 17 (10.0%) weighed ≤ 2500, ≤ 1500, and ≤ 1000 g, respectively. In total, 37 (21.8%) and 12 (7.1%) neonates had GAs ≤ 32 and ≤ 28 weeks, respectively. The median BW was 2600 (IQR: 1716–3235) g, and the median GA was 37.5 (IQR: 32.8–39.0) weeks. The top five etiologies of severe thrombocytopenia in this study were sepsis (65 [38.2%] neonates), genetic defects (26 [15.3%] neonates), neonatal asphyxia (18 [10.6%] neonates), neonatal alloimmune thrombocytopenia (12 [7.1%] neonates), and NEC (11 [6.5%] neonates). Sepsis was the most common cause of thrombocytopenia; 18 (27.7%) and 47 (72.3%) patients had early- and late-onset severe thrombocytopenia, respectively.

**Fig. 1:**
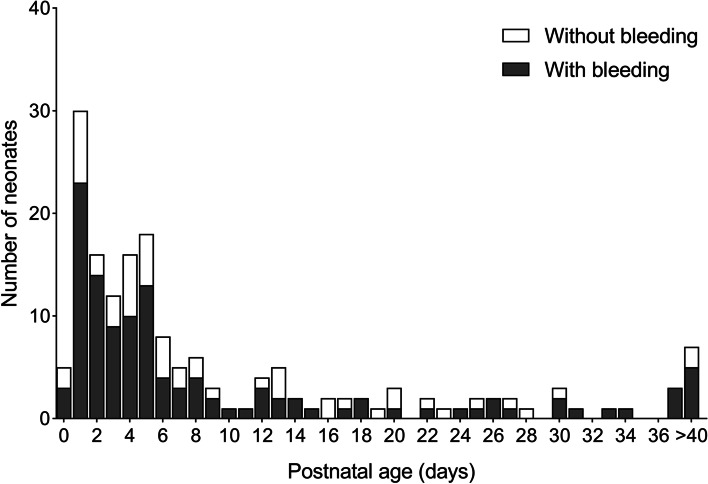
The postnatal age on which each neonate with and without bleeding that severe thrombocytopenia was first recognized

Neonates were divided into two groups according to their clinical symptoms with or without bleeding: 118 (69.4%) with bleeding and 52 (30.6%) without bleeding. Bleeding was observed in 49 (point estimate: 77.8%, 95% CI: 67.2%–88.3%) and 69 (point estimate: 64.5%, 95% CI: 55.3%–73.7%) early- and late-onset severe thrombocytopenic neonates, respectively. An overview of the postnatal age at which severe thrombocytopenia was first recognized in neonates with and without bleeding is shown in Fig. 1. More than one-third of all the neonates presented with severe thrombocytopenia in the first 3 days after birth. Among neonates with bleeding, 42% were detected in the first 3 days, 50% by day 4, and 75% by day 12. Among neonates without bleeding, 27% were found in the first 3 days, 50% by day 6, and 75% by day 16.

### Bleeding

The patterns of the observed bleeding are shown in Table 1. Overall, approximately one-third of the neonates (47 [27.6%]) had more than one type of bleeding. Neonates with the lowest platelet count ≤ 30 × 10^9^/L had a higher incidence rate of bleeding (77.3%) than those with the lowest count between 31 and 50 × 10^9^/L (55.0%). There was a remarkable association between a remarkably low platelet count and cutaneous bleeding (point estimates with 95% CI: 53.7% [44.2%–63.1%] vs. 23.4% [12.3%–34.4%]). Among the 18 neonates with pulmonary hemorrhage (9 neonates ≤ 32 weeks, 3 neonates between 32 and 37 weeks, and 6 neonates > 37 weeks of GA), 8 had NRDS. All neonates with pulmonary hemorrhage and NRDS received mechanical ventilatory support. The mortality rate of neonates with pulmonary hemorrhage was 83.3%. IVH, the most frequent type of ICH, was observed in 22.7% (25/110) and 11.7% (7/60) of neonates with very severe and severe thrombocytopenia, respectively. The incidence of other types of bleeding was similar among neonates with different degrees of thrombocytopenia.

**Table 1:** Bleeding conditions in 170 neonates with severe thrombocytopenia [n(%)]

Bleeding condition	Lowest platelet count (× 10^9^/L)	
	≤ 30 (*n* = 110)	31–50 (*n* = 60)
Extracranial hemorrhage	72(65.5)	23(38.3)
Cutaneous bleeding	59(53.6)	14(23.3)
Umbilical bleeding	7(6.4)	3(5.0)
Pulmonary hemorrhage	13(11.8)	5(8.3)
Gastrointestinal hemorrhage	17(15.5)	7(11.7)
Intracranial hemorrhage	36(32.7)	17(28.3)
Subdural hemorrhage	11(10.0)	6(10.0)
Subarachnoid hemorrhage	5(4.5)	2(3.3)
Solitary (non-cerebellar) parenchymal hemorrhage	5(4.5)	3(5.0)
Cerebellar parenchymal hemorrhage	3(2.7)	1(1.7)
IVH (All Grades)	25(22.7)	7(11.7)
IVH (III/IV)	9(8.2)	3(5.0)

*IVH* Intraventricular hemorrhage

To describe this in more detail, patients were further subdivided into three groups based on the severity of bleeding: major, minor, and no bleeding. The baseline characteristics of the three subgroups are shown in Table 2. The GA of the major bleeding group was the lowest among these groups (the median GAs of the three groups were 38.0 [IQR: 33.1–39.5], 37.8 [IQR: 34.6–38.9], and 36.2 [IQR: 31.4–39.0] weeks, respectively). The BW of the major bleeding group (median: 2310 [IQR: 1213–3210] g) was lower than those of the no bleeding (median: 2495 [IQR: 1728–3071] g) and minor bleeding (median: 2780 [IQR: 2050–3403] g) groups. The comorbidities and treatment of neonates with severe thrombocytopenia are listed in Table 3. Neonates with bleeding (either major or minor) received mechanical ventilation and PT. To investigate the association between nadir platelet count and bleeding, we compared the clinical data of neonates with and without bleeding. Binary logistic regression analysis was used to control for the following confounding factors: GA, BW, PDA, mechanical ventilation, and coagulation disorders. The analysis indicated that a lower minimum platelet count (OR: 2.504 [95% CI: 1.180–5.314], *P* = 0.017) was associated with a higher incidence of bleeding in neonates (Table 4).

**Table 2:** Maternal/ neonatal characteristics of severe thrombocytopenic neonates, divided into subgroups by severity of bleeding [n(%)]

Variables	No bleeding (*n* = 52)	Minor bleeding (*n* = 58)	Major bleeding (*n* = 60)
Multiple pregnancy	11(21.2)	6(10.3)	13(21.7)
Vaginal delivery	20(38.5)	20(34.5)	26(43.3)
PROM > 24 h	7(13.5)	1(1.7)	9(15.0)
Maternal diseases			
Diabetes	7(13.5)	8(13.8)	11(18.3)
Preeclampsia	3(5.8)	4(6.9)	6(10.0)
Thrombocytopenia in pregnancy	4(7.7)	3(5.2)	1(1.7)
Sex (M)	33(63.5)	42(72.4)	36(60.0)
GA (weeks)			
≤ 28	1(1.9)	2(3.4)	9(15.0)
28–32	9(17.3)	6(10.3)	10(16.7)
32–37	13(25.0)	14(24.1)	15(25.0)
> 37	29(55.8)	36(62.1)	26(43.3)
BW (gram)			
≤ 1000	2(3.8)	4(6.9)	11(18.3)
1000–1500	9(17.3)	4(6.9)	5(8.3)
1500–2500	15(28.8)	16(27.6)	16(26.7)
> 2500	26(50.0)	34(58.6)	28(46.7)
Asphyxia	8(15.4)	7(12.1)	15(25.0)
Apgar score < 7 at 1 min	10(19.2)	9(15.5)	17(28.3)
Apgar score < 7 at 5 min	4(7.7)	5(8.6)	9(15.0)

*PROM* Premature rupture of membrane, *M* male, *GA* Gestational age, *BW* Birth weight

**Table 3:** Comorbidities and treatment of severe thrombocytopenic neonates in the three subgroups [n(%)]

Variables	No bleeding (*n* = 52)	Minor bleeding (*n* = 58)	Major bleeding (*n* = 60)
PDA	19(36.5)	22(37.9)	37(61.7)
BPD	3(5.8)	4(6.9)	5(8.3)
NRDS	7(13.5)	8(13.8)	18(30.0)
Shock	5(9.6)	15(25.9)	20(33.3)
Coagulation disorders	16(30.8)	29(50.0)	40(66.7)
Sepsis	31(59.6)	37(63.8)	32(53.3)
NEC	8(15.4)	1(1.7)	8(13.3)
Mechanical ventilation	11(21.2)	22(37.9)	33(55.0)
PT	10(19.2)	25(43.1)	22(36.7)

*PDA* Patent ductus arteriosus, *BPD* Bronchopulmonary dysplasia, *NRDS* Neonatal respiratory distress syndrome, *NEC* Necrotizing enterocolitis, *PT* Platelet transfusion

**Table 4:** Regression analysis for factors associated with bleeding

Variables	OR	95% CI for OR		*P* value
		Lower	Upper	
The minimum platelet count	2.504	1.180	5.314	0.017
GA	0.979	0.955	1.004	0.094
BW	1.001	1.000	1.002	0.005
PDA	1.513	0.577	3.967	0.400
Mechanical ventilation	2.231	0.834	5.969	0.110
Coagulation disorders	2.632	1.120	6.184	0.026

*OR* Odds ratio, *CI* Confidence interval, *GA* Gestational age, *BW* Birth weight, *PDA* Patent ductus arteriosus

For the minimum platelet count, using a platelet count of 30 × 10^9^/L as the cut-off to convert to categorical data. Reference category is platelet count 31–50 × 10^9^/L group

**Fig. 2:**
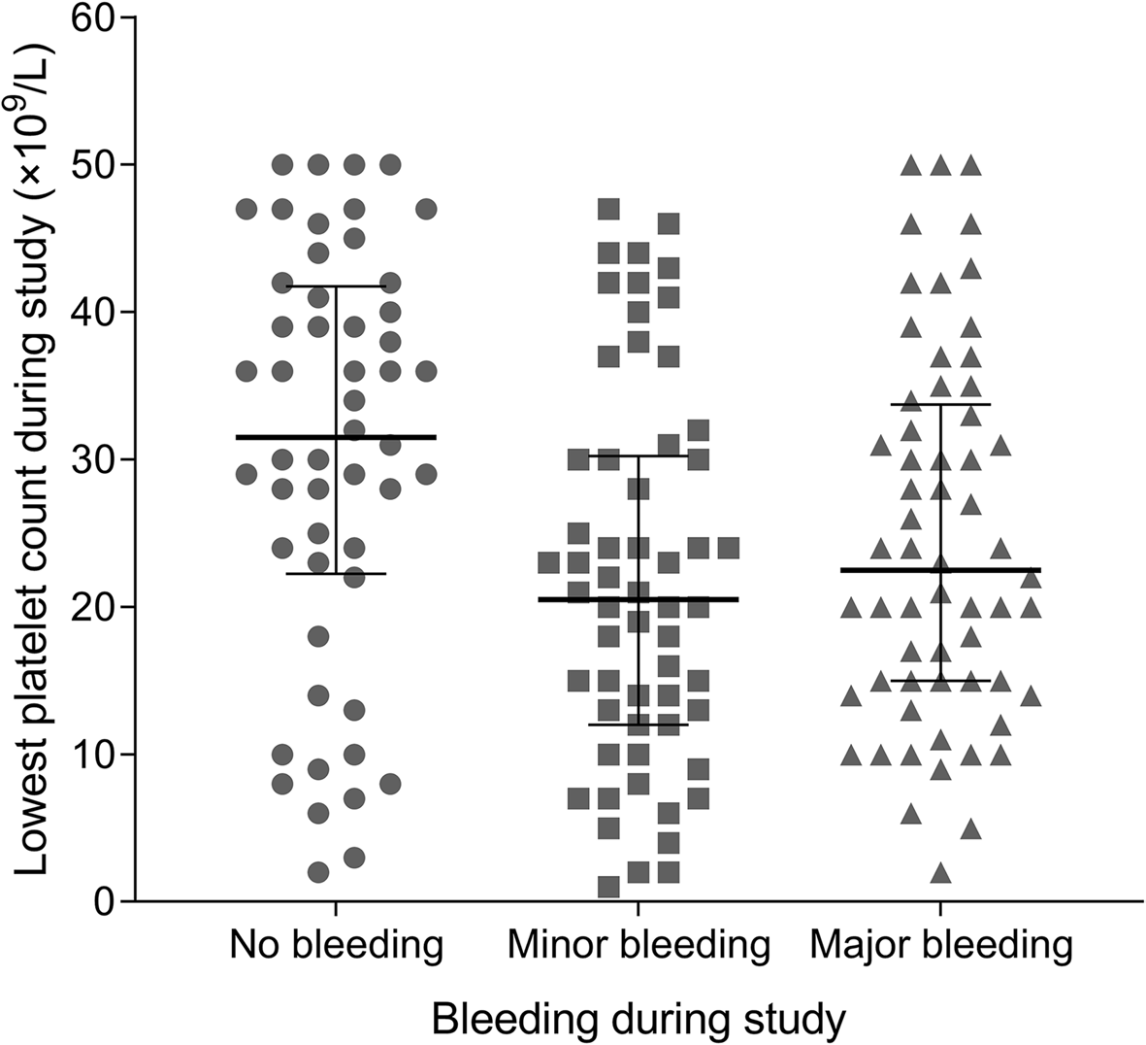
The lowest platelet counts in neonates with and without bleeding during study. Lines represent median with interquartile ranges

Figure 2 shows the lowest platelet counts of neonates during the study in the three subgroups. The minimal platelet counts were lower in both the minor bleeding (point estimate: 21.9 × 10^9^/L, 95% CI: 18.5–25.2 × 10^9^/L) and major bleeding (point estimate: 24.6 × 10^9^/L, 95% CI: 21.3–27.8 × 10^9^/L) groups than in the no bleeding group (point estimate: 30.4 × 10^9^/L, 95% CI: 26.4–34.3 × 10^9^/L).

### Platelet transfusion

In total, 57 (33.5%) of the 170 neonates received ≥ 1 PT (median: 1 [range: 1–9]). More than half of the initial transfusions were ordered before postnatal 2 weeks. Among those with a minimum platelet count between 31 and 50 × 10^9^/L, only 3 (5%) neonates received PT in unstable conditions. Half of those with a minimum platelet count ≤ 30 × 10^9^/L received a transfusion. Very severe thrombocytopenia was significantly associated with a higher rate of PT (point estimates with 95% CI: 49.1% [39.6%–58.6%] vs. 5.7% [0.7%–10.7%]). The total number of PTs administered was higher in neonates with bleeding (IQR: 0–1 [range: 0–9] transfusion) than in those without bleeding (IQR: 0 [range: 0–6] transfusion). Of the 113 neonates with no PT, 23 (20.4%) died due to other underlying causes.

### Outcomes

The median inpatient length of stay was 14.0 days: 15.0 and 13.0 days for the group without and with bleeding, respectively. Thrombocytopenia resolved in 76 (44.7%) neonates and persisted in 51 (30.0%) neonates at discharge. In total, 43 (25.3%) neonates died in the overall study group: 10 (point estimate: 15.9%, 95% CI: 6.6%–25.2%) and 33 (point estimate: 30.8%, 95% CI: 21.9%–39.7%) with early- and late-onset severe thrombocytopenia, respectively. The top three causes of death were multiple organ failure (7 [16.3%] neonates), respiratory failure (6 [14.0%] neonates), and disseminated intravascular coagulation (5 [9.4%] neonates). Only 3 neonates directly died of bleeding (2 for IVH and 1 for pulmonary hemorrhage). The platelet count of at least 38 (88.4%) neonates was still < 150 × 10^9^/L at death. The mortality rate was remarkably related to bleeding conditions in 39 (point estimate: 33.1%, 95% CI: 24.4%–41.7%) neonates, and 4 (point estimate: 7.7%, 95% CI: 0.2%–15.2%) neonates without bleeding died. The major bleeding group (27 [45.0%] neonates) had 5 times higher mortality rate than the no bleeding group (4 [7.7%] neonates). In addition, neonates with very severe thrombocytopenia (point estimate: 29.1%, 95% CI: 20.5%–37.7%) had a comparable mortality rate to those with severe thrombocytopenia (point estimate: 18.3%, 95% CI: 8.3%–28.4%).

## Discussion

Severe thrombocytopenia is a rare but life-threatening condition in the NICU [15]. To date, only a few studies have examined this important topic [4–6, 10, 11]. In the present study, severe thrombocytopenia was reported in 194 of 5819 neonates who had been admitted to our NICU for the last 5 years. The incidence rate of severe thrombocytopenia was 3.33% among all hospitalized neonates, which was in line with the rates reported in previous studies [3–5]. GA and BW were not negatively associated with the incidence of severe thrombocytopenia, as mentioned in previous studies [4, 5, 11]. Sepsis is the most frequent cause of thrombocytopenia, as reported in several studies [16, 17].

The higher incidence of total hemorrhage in neonates with severe thrombocytopenia discovered in our study was similar to that reported in the literature [10]. A previous study revealed that early-onset severe thrombocytopenia was implicated in an increased number of bleeding events; a similar result was observed in our study [10]. Some neonates experienced multiple types of bleeding, including ECH and ICH, and the percentage of individual events failed to add up to 100%, as listed in Table 1. We included some forms of hemorrhage that were not frequently observed in other studies, such as umbilical bleeding and ICH (non-intraventricular). We did not record hematuria because dipstick urinalysis is not routinely performed in our NICU [18].

Cutaneous bleeding with petechiae, ecchymosis, or oozing from venipuncture sites is highly common in neonates with severe thrombocytopenia [5, 17]. We observed that this type of bleeding was recorded in 53.6% of neonates with a nadir platelet count of ≤ 30 × 10^9^/L, compared with 23.3% of those with a nadir count between 31 and 50 × 10^9^/L. Our cutaneous bleeding rate was higher than those reported by Baer et al. [5] and Muthukumar et al. [10]: 42.9% compared with 11.7% and 39.1%, respectively. In contrast, the lower incidence of cutaneous bleeding in Baer et al.’s [5] study may be due to the strict definition of heavy bruising (not petechial only) or oozing from venipuncture sites. The incidence rate of IVH in neonates with very severe thrombocytopenia (point estimate: 22.7%, 95% CI: 14.8%–30.7%) was similar to that in neonates with severe thrombocytopenia (point estimate: 11.7%, 95% CI: 3.3%–20.0%) [5, 19]. However, the trend that lower platelet counts were correlated with a higher incidence of IVH was consistent with the findings of Bolat et al. [7] and Saber et al. [16]. However, it remains uncertain whether thrombocytopenia causes IVH before the occurrence of thrombocytopenia [4, 5]. In our subsequent studies, a deeper inquiry should be conducted to determine whether there was a causal association between severe thrombocytopenia and IVH. Other types of bleeding were also described, and we found no evident connection between bleeding and the severity of severe thrombocytopenia. This suggests that pathological hemorrhage in neonates is multifactorial and unlikely to be attributable solely to a lower platelet count [20].

To better explore bleeding in neonates with severe thrombocytopenia, we obtained neonates in groups of three according to the severity of bleeding: no, minor, and major bleeding. We found that the GA and BW of neonates who developed major bleeding were lower than those of neonates without bleeding. Neonates with no bleeding had the highest nadir platelet counts among the three subgroups, which was in contrast to the results of previously published studies [4, 11]. One reason was that the definition of major bleeding was more common in our study; gastrointestinal hemorrhage and ICH (non-intraventricular) were also included. Second, Stanworth et al. [4] recorded the minimum counts before bleeding proactively, whereas we defined different types of bleeding at the end of the study and considered records as the nadir platelet counts prior to bleeding events, as in Fustolo-Gunnink et al.’s study [11].

This study revealed that bleeding occurred more frequently in neonates with lower minimum platelet count (point estimates with 95% CI: 77.3% [69.3%–85.2%] vs. 55.0% [42.0%–68.0%]). A significant association was observed after adjusting for possible confounding factors. We also performed additional statistical analyses to determine whether the minimal platelet count had sufficient power (with a probability of 0.829) to show its association with bleeding in our study. We hypothesized that a lower minimum platelet count played a more important role in cutaneous bleeding and IVH. We also found that BW and coagulation disorders were associated with bleeding. However, GA had no association with bleeding, which could be explained by the small sample size, which weakened the strength of the evidence in our study. Subsequently, we plan to conduct a multicenter study to address this problem.

PT has long been considered a common therapeutic or preventative measure to reduce bleeding in neonates with severe thrombocytopenia; however, it is also associated with risks [4, 17]. Several studies have demonstrated that an increased number of PTs are associated with a higher mortality risk [5, 7, 16, 21–24]. The rate of PT varies from 39 to 72% in neonates with severe thrombocytopenia in different NICUs [7, 16, 22, 22, 23]. In our study, one or more PTs were administered to 57 (33.5%) neonates with severe thrombocytopenia. Neonates with bleeding received more PTs than those without bleeding did. We hypothesized that patients with bleeding required more PTs or experienced deleterious events of excessive PTs than those without bleeding. We did not administer PT to every neonate with very severe thrombocytopenia, as suggested by the guidelines. On the one hand, during the time waiting for platelet application approval, the platelet count increased to over 30 × 10^9^/L and failed to reach indications for PTs. On the other hand, in clinical practice, doctors will combine patients’ clinical symptoms and laboratory tests to determine the use of PT because we believe that the use of PT should be stricter unless the conditions worsen. Therefore, we cannot easily assess the association between PT and death because it is uncertain whether the death was caused by patients in poor conditions receiving more PTs or adverse effects of the transfusion process.

The duration of hospital stay was not affected by bleeding status or severity of severe thrombocytopenia. Other factors were more prominent during hospitalization than those mentioned previously. The mortality rate was 25.3% for all neonates. Neonates with late-onset severe thrombocytopenia had a higher mortality rate than those with early-onset severe thrombocytopenia (30.8% vs. 15.9%). This can be explained by the fact that severe thrombocytopenia occurring ≥ 72 h after birth is secondary to sepsis and/or NEC and has more serious and life-threatening conditions [3, 8]. We found comparable mortality rates among neonates with different degrees of severe thrombocytopenia. The studies by Baer et al. [5] and Bolat et al. [7] arrived at the same conclusion as we did.

This study has some limitations. A single-center retrospective design might have had an impact on the completeness of data collection and follow-up of patients. Meanwhile, in the presence of confounding factors, clinical data must be interpreted with caution. For example, we cannot specifically confirm when bleeding occurs and the minimum platelet count before PT because the related data were acquired during daily routine clinical practice. As a result, the cause of PT and severe thrombocytopenia or bleeding could not be easily evaluated. The morbidity of gastrointestinal hemorrhage is probably underestimated because we excluded patients who underwent surgery, a quarter of whom had colostomy after NEC. Patient data, such as intrauterine growth restriction and antenatal steroids, were not included because several patients were treated in the outer court before being transferred to our specialized children’s hospital.

## Conclusions

Taken together, we described the incidence of severe thrombocytopenia and found that bleeding was very common in neonates with severe thrombocytopenia. A low minimum platelet count was associated with bleeding. The role of PT in the prevention and treatment of bleeding events deserves further exploration in clinical practice. In addition, the advantages and disadvantages should be carefully balanced before applying PTs in the NICU.

## Supplementary Information

**Additional file 1.**

**Additional file 2.**

## Data Availability

All data generated or analysed during this study are included in this published article and its supplementary information files.

## Abbreviations

BW: Birth weight
CI: Confidence interval
ECH: Extracranial hemorrhage
GA: Gestational age
ICH: Intracranial hemorrhage
IQR: Interquartile range
IVH: Intraventricular hemorrhage
NEC: Necrotizing enterocolitis
NICU: Neonatal intensive care unit
NRDS: Neonatal respiratory distress syndrome
ORs: Odds ratios
PDA: Patent ductus arteriosus
PT: Platelet transfusion
