# Supplementary material for: Bleeding in neonates with severe thrombocytopenia: a retrospective cohort study
Source: BMC Pediatr. 2022 Dec 22;22:730. doi: 10.1186/s12887-022-03802-4 (PMC9773444; doi:10.1186/s12887-022-03802-4)
Supplement: Supplementary file 2 — Additional file 2. [file 12887_2022_3802_MOESM2_ESM.docx]

**Supplement**

In our study, only 2 (1.2%, 2/170) observations were missing for the variable of number of platelet transfusion. Missing data imputation was based on means of the number of platelet transfusion in the study population. We have marked the missing values in yellow in the Data sheet.
